# Isolation and characterization of a lectin from *Andira anthelmia *seeds

**DOI:** 10.1186/1753-6561-8-S4-P238

**Published:** 2014-10-01

**Authors:** Cleane Moreira, Mayara Silva, Camila Nobre, Thaiz Miguel, Antonia Nascimento, Celso Nagano, Kyria Nascimento, Benildo Cavada, Ivanice Silva

**Affiliations:** 1 Universidade Federal do Ceará, Fortaleza, CE, Brazil; 2 Universidade Regional do Cariri, Crato, CE, Brazil

## 

*Andira anthelmia *seeds, a species belonging to the Leguminosae family, Papilionoideae subfamily, Dalbergieae tribe, have a glucose/mannose specific lectin that agglutinates rabbit erythrocytes treated with trypsin. The Dalbergieae tribe has lectins which have specificity for different carbohydrates including mannose and also have several biological activities such as induction of rat paw edema, release of chemotactic mediators by macrophages, vasorelaxant effect in rat aortas, termiticide activity, potential fungicide action, among others. This study aimed to isolate, purify and physicochemically characterize a lectin found in seeds of *Andira anthelmia*. The lectin from *Andira anthelmia *seeds was purified by affinity chromatography on Mannose-Sepharose matrix followed by ion exchange chromatography on DEAE-Sephacel matrix. This procedure resulted in a purified lectin, named AAL. AAL purification process was monitored by specific hemagglutinating activity and SDS-PAGE, in which it was observed that this lectin has a molecular weight of approximately 20 kDa and four others subunits of approximately 15 and 14 kDa. This lectin is a glycoprotein with approximately 1.89% of carbohydrates on its composition and shows high stability, being able to maintain their hemagglutinating activity in a wide pH range and after exposure to temperatures of 70 °C for one hour. After dialysis against the chelating agent EDTA, AAL lost its hemagglutinating activity, but recovered its action after the addition of metals, being, therefore, dependent on divalent metal cations. In this study a new lectin from Dalbergieae was purified and characterized. Further analyzes are needed in order to best evaluate their biotechnological applications.

